# Untargeted metabolomics of the cochleae from two laryngeally echolocating bats

**DOI:** 10.3389/fmolb.2023.1171366

**Published:** 2023-04-19

**Authors:** Hui Wang, Ruyi Sun, Ningning Xu, Xue Wang, Mingyue Bao, Xin Li, Jiqian Li, Aiqing Lin, Jiang Feng

**Affiliations:** ^1^ College of Life Science, Jilin Agricultural University, Changchun, China; ^2^ Jilin Provincial Key Laboratory of Animal Resource Conservation and Utilization, Northeast Normal University, Changchun, China

**Keywords:** bats, cochlea, echolocation, high-frequency hearing, untargeted metabolomics

## Abstract

High-frequency hearing is regarded as one of the most functionally important traits in laryngeally echolocating bats. Abundant candidate hearing-related genes have been identified to be the important genetic bases underlying high-frequency hearing for laryngeally echolocating bats, however, extensive metabolites presented in the cochleae have not been studied. In this study, we identified 4,717 annotated metabolites in the cochleae of two typical laryngeally echolocating bats using the liquid chromatography–mass spectroscopy technology, metabolites classified as amino acids, peptides, and fatty acid esters were identified as the most abundant in the cochleae of these two echolocating bat species, *Rhinolophus sinicus* and *Vespertilio sinensis*. Furthermore, 357 metabolites were identified as significant differentially accumulated (adjusted *p*-value <0.05) in the cochleae of these two bat species with distinct echolocating dominant frequencies. Downstream KEGG enrichment analyses indicated that multiple biological processes, including signaling pathways, nervous system, and metabolic process, were putatively different in the cochleae of *R. sinicus* and *V. sinensis*. For the first time, this study investigated the extensive metabolites and associated biological pathways in the cochleae of two laryngeal echolocating bats and expanded our knowledge of the metabolic molecular bases underlying high-frequency hearing in the cochleae of echolocating bats.

## 1 Introduction

Echolocation is a remarkable and perceptive behavior that is well evolved in bats, which is usually used for orientation, obstacle avoidance, and hunting (Jones, 2005; Jones and Teeling, 2006). Although echolocation is found in several mammalian lineages (He et al., 2021), laryngeally echolocating bats are renowned for their sophisticated echolocation (Simmons et al., 1979; Jones and Teeling, 2006). High-frequency hearing is an important component of echolocation and is essential for echolocating bats to perceive ultrasonic signals (Wohlgemuth et al., 2016; Moss, 2018). The molecular bases underlying echolocation accompanied by high-frequency hearing have attracted increasing attentions (Cao et al., 2022).

The majority of echolocating bats are usually referred to as laryngeally echolocating bats who can emit ultrasonic vocalizations through their larynxes (Waters and Vollrath, 2003; Yovel et al., 2010), including constant-frequency (CF) bats and frequency-modulated (FM) bats. Therefore, both CF and FM bats have been identified as laryngeally echolocating bats in the true sense of the term with high-frequency hearing. The realization of high-frequency hearing involves many organs and physiological processes, among them, the cochlea is a most important organ of the auditory system (Adams and Pedersen, 2013; Moss, 2018). It is a snail-shaped inner ear structure that plays important roles in sound perception, signal processing, and transmission to the brain (Davies et al., 2013; Moss, 2018). Previous studies have demonstrated that the cochleae of laryngeally echolocating bats have possessed special structural, physiological and genetic adaptations for detecting high-frequency acoustic signals (Ulanovsky and Moss, 2008; Schnitzler and Denzinger, 2011; Vater and Kössl, 2011; Davies et al., 2013).

Transcriptomic approaches have been usually used to uncover candidate genes and biological pathways underlying the genetic bases of adaptations for high-frequency hearing in echolocating bats (Dong et al., 2013; Wang et al., 2018; Ma et al., 2020). A study involving comparative inner ear transcriptomic analysis between *M. ricketti* (FM echolocating bat) and *Cynopterus sphinx* (non-echolocating bat) demonstrated that the genes upregulated in *Myotis ricketti* were particularly associated with cochlear morphogenesis, inner ear morphogenesis, and sensory perception of sound categories, which are consistent with the morphological and physiological differentiation of the inner ear between these two species (Dong et al., 2013). In addition, comparative cochlear transcriptomic analyses of four different bat species have demonstrated variations of gene expression among the bats and different nervous system activities during auditory perception in the cochlea particularly in CF bats (Wang et al., 2018). Besides, numerous efforts to identify high-frequency hearing-related genes have examined differences in coding sequences between echolocating and non-echolocating mammalian species including bats and even whales (Li et al., 2008; Liu et al., 2010; Davies et al., 2012; Wang et al., 2020; Liu et al., 2022). Genome-wide screening has also revealed that multiple hearing-related genes show molecular adaptation in lineages of echolocators (Parker et al., 2013; Zou and Zhang, 2015; Liu et al., 2018; Liu et al., 2022).

In recent years, an accumulating body of researches have indicated that abundant metabolites may be particularly important for specific phenotype (Keurentjes, 2009; Zampieri and Sauer, 2017; Han et al., 2021). Metabolomics is a new branch of “-omics” science in the postgenomic era that has high potential due to its close relationship with phenotype (Fraga-Corral et al., 2022). In addition to the hearing-related genes, cochlear metabolites may also play important roles in the realization of high-frequency hearing for echolocating bats (Meng et al., 2020; Wörheide et al., 2021). However, cochlear metabolites and associated metabolic pathways in laryngeally echolocating bats have been less clearly understood.

Therefore, in this study, we explored the utility of liquid chromatography–mass spectroscopy (LC-MS) to take an insight into the cochlear metabolites of two typical laryngeally echolocating bats, *R. sinicus* and *V. sinensis*. *R. sinicus*, belonging to the Rhinolophidae, is a typical CF bat species, whose dominant frequency is around 83.15 kHz (Xie et al., 2017; Wang et al., 2018). Meanwhile, *Vespertilio sinensis* is a typical FM bat and belongs to the Vespertilionidae, with a dominant frequency around 24.2 kHz (Fukui et al., 2004). For the first time, we here aim to detect the metabolites responsible for the cochlear function of laryngeally echolocating bats. We also aim to uncover the differences of metabolites and related biological processes in the cochleae between these two bat species. This study is expected to provide a new perspective for the studies of echolocation and high-frequency hearing in bats.

## 2 Materials and Methods

### 2.1 Sample collection

Six biological repeats for each bat species were included for the metabolomic analyses. Therefore, six adults of *R. sinicus* and *V. sinensis* were captured during July, respectively. To avoid any influence of sex-related differences, only females were selected for inclusion in the study. All individuals were euthanized by cervical dislocation, and a pair of cochleae from each individual were collected and immediately flash-frozen in liquid nitrogen in the field, before transfer to a −80°C freezer.

### 2.2 Metabolite extraction and sequencing sample preparation

Equal amounts of the cochlear samples from the two bat species (30 mg, n = 12) were transferred to a 2 ml centrifuge tube, supplemented with 600 µl of precooled 50% methanol (stored at −20°C) containing 2-amino-3-(2-chlorophenyl)-propionic acid (4 ppm), and vortexed for 1 min. Subsequently, the samples were precooled at −20°C for 2 min, supplemented with 100 mg of glass beads, and placed in a tissue grinder for 2 min at 60 Hz. Room temperature ultrasonic extraction was then applied for 15 min, followed by storage at −20°C for 30 min. After centrifugation at 13,000 g for 10 min at 4°C, the supernatant was transferred to a new tube. The extraction solution was vacuum-dried and resuspended in 50% methanol (vortexing for 30 s and ultrasonic extraction for 3 min), followed by storage at −20°C for 2 h. Finally, centrifugation was applied at 13,000 g for 10 min at 4°C, and the obtained supernatant was transferred into a detection bottle. Meanwhile, pooled quality control (QC) samples were prepared by mixing an equal volume of each extraction sample. All the samples were stored at −80°C prior to the LC-MS analysis. The experimental process mainly refers to the reagent supplies manual instruction and references (Zhou et al., 2012; Cao et al., 2020).

### 2.3 LC-MS-based metabolomic analysis

All samples were analyzed using an ACQUITY UPLC I-Class system (Waters Corporation, Milford, MA, USA) coupled with a VION IMS QTOF mass spectrometer (Waters Corporation, Milford, MA, United States) for metabolic profiling in both ESI positive and ESI negative ion modes. An ACQUITY UPLC BEH C18 column (1.8 μm, 2.1 × 100 mm) was employed in both positive and negative modes. Water and acetonitrile/methanol 2/3 (v/v), both containing 0.1% formic acid, were used as mobile phases A and B, respectively. The following linear gradient was applied: 0.01 min, 5% B; 4 min, 30% B; 8 min, 50% B; 10 min, 80% B; 14 min, 100% B; 15 min, 100% B; 15.1 min, 5% B; and 16 min, 5% B. The flow rate was 0.35 mL/min and the column temperature was maintained at 45°C. All samples were kept at 4°C during the analysis. The injection volume was 2 μL. The mass range was from m/z 100 to 1,200. The resolution was set at 70,000 for the full MS scans and 17,500 for the HCD MS/MS scans. The collision energy was set at 10, 20, and 40 eV. The mass spectrometer was operated as follows: spray voltage, 3,800 V (+) for the positive ion mode and 3,200 V (−) for the negative ion mode; sheath gas flow rate, 40 arbitrary units; auxiliary gas flow rate, 8 arbitrary units; capillary temperature, 320°C; probe heater temperature, 350°C; and S-lens RF level, 50. The QC samples (created by pooling all of the samples) were injected at regular intervals throughout the analytical run to provide a set of data from which repeatability could be assessed (Zhou et al., 2012; Cao et al., 2020).

### 2.4 Metabolomic data processing

The raw LC-MS data were processed using the software Progenesis QI V2.3 (Non-linear Dynamics, Newcastle, United Kingdom) for baseline filtering, peak identification, integrity, retention time correction, peak alignment, and normalization. The main parameters of 5 ppm precursor tolerance, 10 ppm product tolerance, and 5% product ion threshold were applied. Compound identification was based on the precise mass-to-charge ratio (m/z), secondary fragments, and isotopic distribution using the Human Metabolome Database (HMDB, http://www.hmdb.ca), The Kyoto Encyclopedia of Genes and Genomes (KEGG, http://www.kegg.com/), Lipidmaps (V2.3), Metlin, EMDB, PMDB, and custom-made databases to perform qualitative analysis. The extracted data were then further processed by removing any peaks with a missing value (ion intensity = 0) in more than 50% in groups, and by screening according to the qualitative results of the compound. Compounds with resulting scores below 36 points were also deemed to be inaccurate and removed (the full score is 60 and the pass mark is 36). A data matrix was combined from the positive ion and negative ion data. The matrix was imported into R to carry out principal component analysis (PCA) to observe the overall distribution among the samples and the stability of the whole analytical process. Orthogonal partial least-squares discriminant analysis (OPLS-DA) and partial least-squares discriminant analysis (PLS-DA) were used to distinguish the metabolites that differ between groups. To prevent overfitting, sevenfold cross-validation and 200 response permutation testing (RPT) were used to evaluate the quality of the model. Variable importance of projection (VIP) values obtained from the OPLS-DA model were used to rank the overall contribution of each variable to group discrimination. Two-tailed Student’s t-test was further used to verify that the metabolites differing between groups were significant. Differential metabolites with VIP values greater than 1.0 and *p*-values less than 0.05 were selected.

### 2.5 Differentially accumulated metabolites (DAMs) identified and bioinformatic analysis

To reveal the differences between the cochleae of *R. sinicus* and *V. sinensis*, a series of criteria were used to identify the DAMs among annotated metabolites: VIP >1, |fold change| > 1.5, and adjusted *p*-value <0.05. Visualization of the DAMs in the two bat species was achieved by creating heatmaps and a volcano plot using the OmicShare tools (http://www.omicshare.com/tools), a free online platform for data analysis. Among the DAMs, the predominantly accumulated metabolites in the cochleae of *R. sinicus* and *V. sinensis* were submitted to the OmicShare tools (http://www.omicshare.com/tools) to identify the representative KEGG pathways for further elucidation of the functional properties.

## 3 Results

### 3.1 Global metabolites detected in the cochlea of two laryngeally echolocating bat species

To explore the global metabolites in the cochleae of the two laryngeally echolocating bats, *R. sinicus* and *V. sinensis*, untargeted metabolomic analyses were performed, which identified 4,717 annotated metabolites from 10,958 positive ion and negative ion features (Table 1; Supplementary Table S1). The overlapping total ion chromatograms (TIC) of positive mode and negative mode of all samples and the QC samples demonstrated that all samples obtained in the positive ion and negative ion modes had a good overlap, which indicated that this model was stable, reproducible, and consistent for all of the samples (Supplementary Figure S1).

**TABLE 1:** Basic information of the cochlear metabolomics of the two echolocating bats.

Identification	Metabolites	Annotated metabolites	Super-class	Class	Sub-class
Positive ion	5,867	3,108	2,325	2,323	2,172
Negative ion	5,091	1,609	1,085	1,082	1,005
Total	10,958	4,717	3,410	3,405	3,177

### 3.2 Multivariate statistical analysis of sequencing samples

**FIGURE 1:**
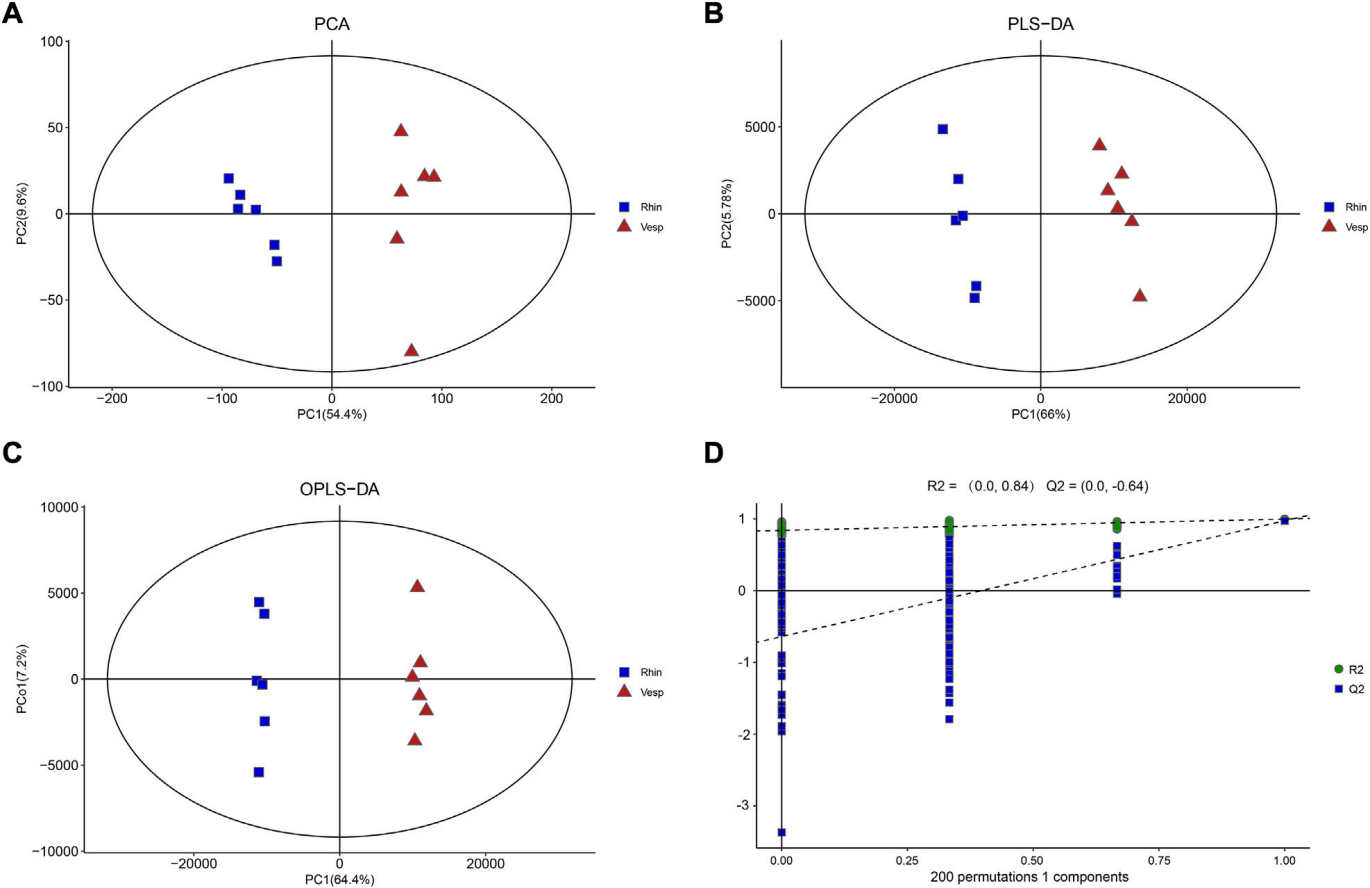
Results of multivariate analysis of cochlear samples from the *R. sinicus*, and *V. sinensis*. **(A)** PCA score plot, **(B)** OPLS-DA score plot, **(C)** PLS-DA score plot, **(D)** permutation plot. There is a clear separation of *R. sinicus*, and *V. sinensis*.

PCA was used to determine the sample separation and aggregation between *R. sinicus* and *V. sinensis* (Figure 1A). Each point on the PCA score graph represents a single sample. Aggregation of points indicates that the observed variables are highly similar, while discrete points represent significant differences in the observed variables. The PCA scores illustrated that PC1 and PC2 were responsible for 54.4% and 9.6% of the variation, respectively, indicating a clear separation between these two bat species. The results demonstrated that *R. sinicus* and *V. sinensis* had different cochlear metabolic characteristics. In addition, PLS-DA, which is a supervised discriminant profiling statistical method, was used to identify more specific differences between the groups (Figure 1B). Accordingly, higher values for PLS-DA model parameters denote greater reliability for the PLS-DA model. R2 of the PLS-DA model was 0.998 and Q2 was 0.967, which denoted greater reliability for the PLS-DA model. According to the PLS-DA model parameters, this model was reasonable for interpreting the differences between the two bat species. In addition, the OPLS-DA score plot demonstrated a clearer separation of *R. sinicus* and *V. sinensis* and the parameters were as follows: R2X = 0.774, R2Y = 0.998, and Q2 = 0.974, indicating that the current OPLS-DA model is more reliable and that consistent modeling and predictability were achieved (Figures 1C, D). Therefore, these data were used for subsequent analyses.

### 3.3 Classification of metabolites

**FIGURE 2:**
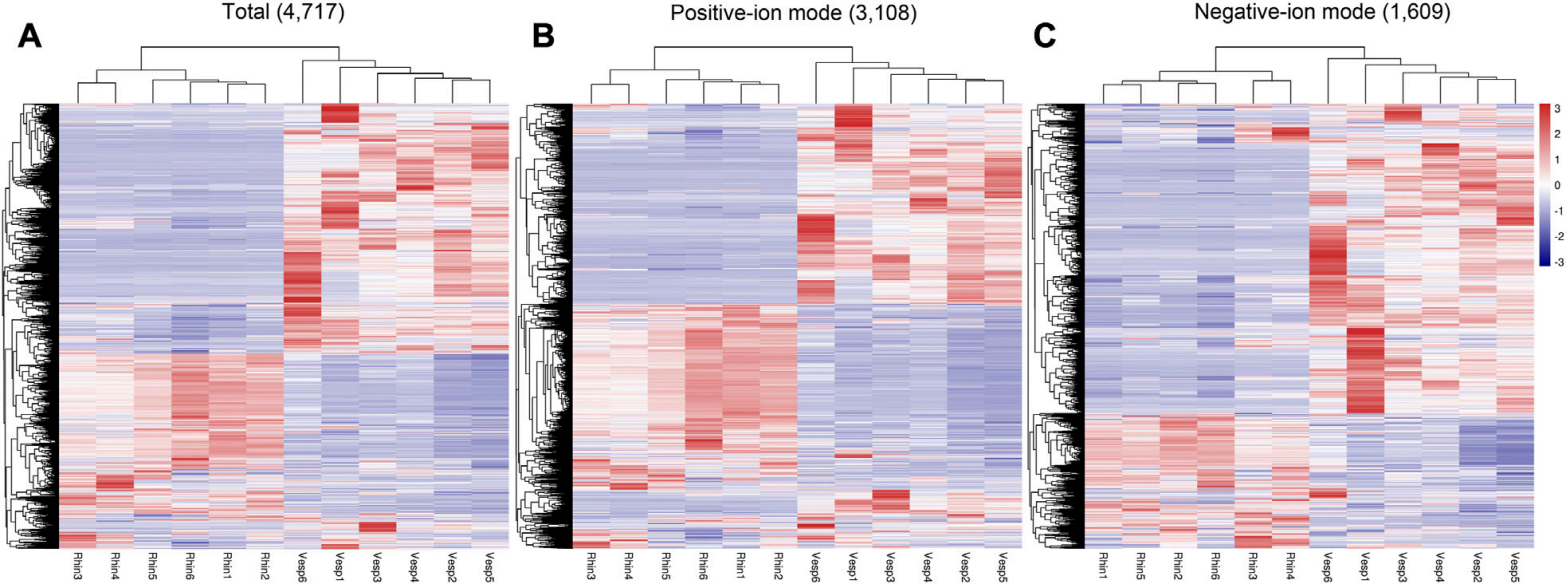
Heatmaps of the 4,717 annotated metabolites identified in the cochlea of *R. sinicus* and *V. sinensis*. **(A)** Total ions, **(B)** Positive ion mode, **(C)** Negative ion mode. Rhin and Vesp stand *R. sinicus* and *V. sinensis*, respectively, as also used elsewhere in this paper.

In addition, heatmaps of the 4,717 annotated metabolites identified in the cochleae of the two echolocating bats are presented in Figure 2, which illustrates that metabolites varied greatly between *R. sinicus* and *V. sinensis* according to the total metabolites and both sets of metabolites detected based on positive ion and negative ion analyses.

**FIGURE 3:**
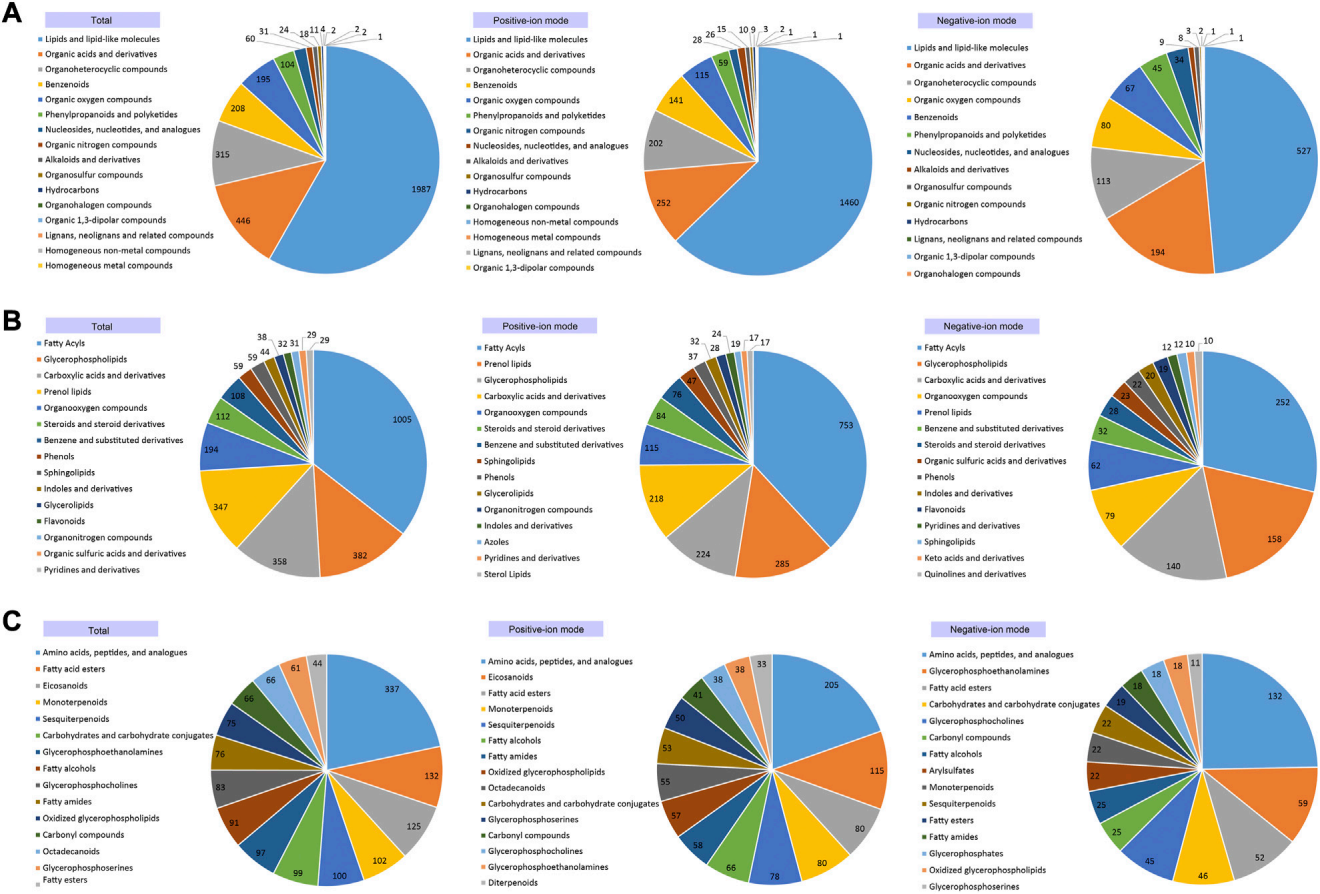
Annotated cochlear metabolites detected for Super-classification **(A)**, Classification **(B)** and Sub-classification **(C)**, respectively.

Based on the annotations of the 4,717 metabolites, most metabolites were assigned to at least one metabolic category and various different types of metabolites were detected in the cochleae of the two echolocating bats (Supplementary Table S2). Among these 4,717 annotated metabolites, 3,410 metabolites, 3,405 metabolites, and 3,177 metabolites were identified to be Super-class, Class, and Sub-class metabolic categories (Figure 3; Supplementary Table S2). At the Super-class level, the three largest metabolic categories were Lipids and lipid-like molecules (1,987 metabolites), Organic acids and derivatives (446 metabolites), Organoheterocyclic compounds (315 metabolites) (Figure 3A). Similarly, metabolites detected at the Class and the Sub-class levels were illustrated by Figures 3B, C, respectively.

**FIGURE 4:**
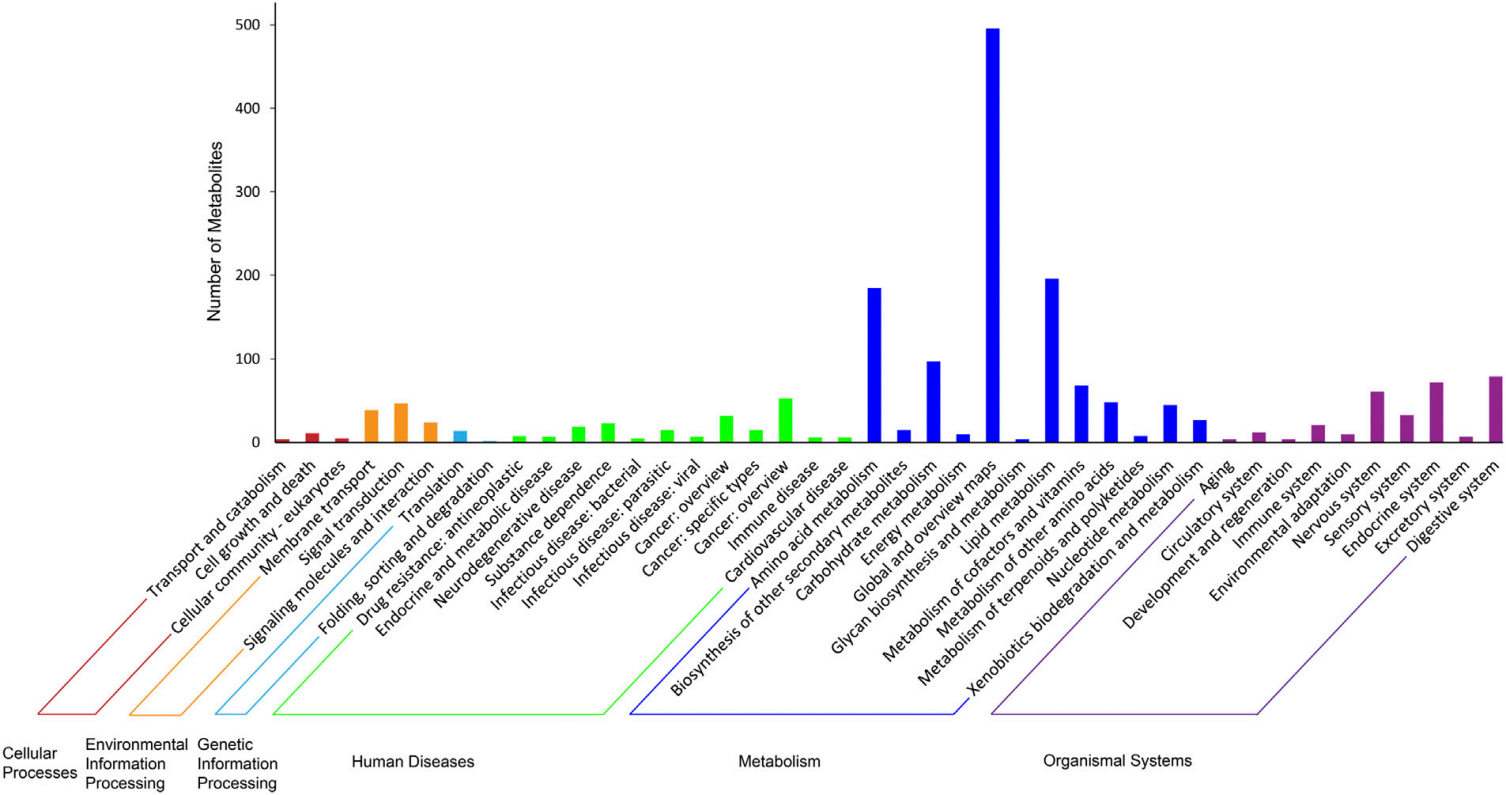
The KEGG categories of all identified metabolites.

Various metabolic categories were identified for the metabolites detected in the cochleae of the two echolocating bats. Figure 4 has showed the KEGG classes of all identified metabolites, indicating that several metabolism related processes were the most abundant, including Amino acid metabolism, Lipid metabolism, and Carbohydrate metabolism. Further analysis of the third hierarchical levels of KEGG pathways is listed in Supplementary Table S3. The top 5 largest KEGG pathways were Metabolic pathways (416), Arachidonic acid metabolism (46), Glycerophospholipid metabolism (39), Biosynthesis of amino acids (39), and ABC transporters (39).

### 3.4 DAMs detected in the cochlea between *R. sinicus* and *V. sinensis*

**FIGURE 5:**
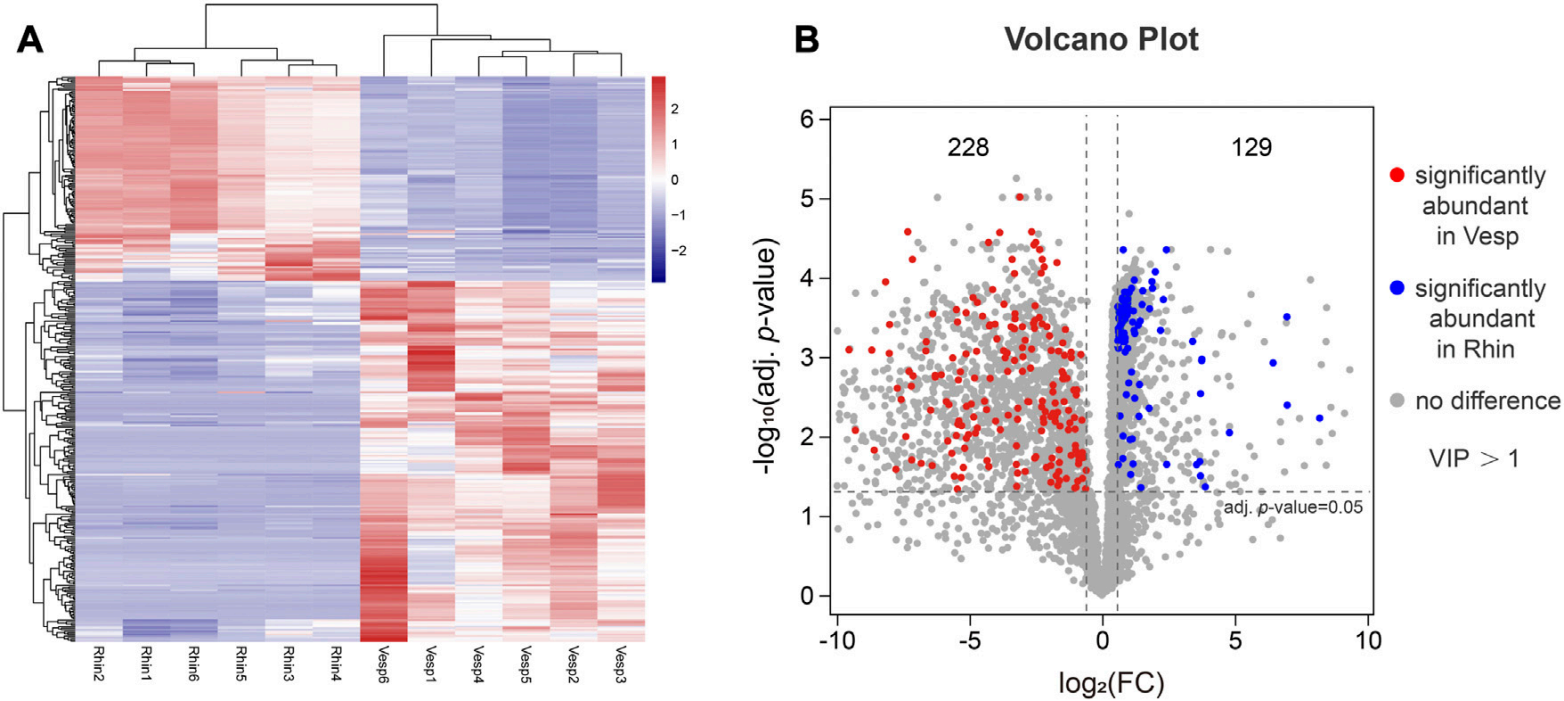
Visualization of DAMs present at significantly different levels in *R. sinicus* and *V. sinensis*. **(A)** Heatmaps of DAMs with the abundance levels in all cochlear samples. Red indicates an increase, blue indicates a decrease, rows indicate different metabolites, and columns indicate different samples. **(B)** Volcano plot of DAMs.

Accordingly, 4,717 high-quality metabolites were used to screen the significant DAMs using the criteria referred to in the Materials and Methods. A total of 357 DAMs were identified to be significantly differentially accumulated (adjusted *p*-value <0.05) between *R. sinicus* and *V. sinensis*, including 129 and 228 metabolites significantly abundant in the cochleae of *R. sinicus* and *V. sinensis*, respectively. A heatmap was used to exhibit the 357 DAMs with different levels in the cochleae of *R. sinicus* and *V. sinensis* (Figure 5A), which suggested that significantly different metabolites were accumulated in the cochleae of the two bat species. A volcano plot indicated the results of analyzing the significance of the DAMs between *R. sinicus* and *V. sinensis* (Figure 5B). Concretely, the DAMs were further assigned to the Super-class (Supplementary Figure S2), Class (Supplementary Figure S3), and Sub-class categories (Supplementary Figure S4) indicating variations of cochlear metabolites between *R. sinicus* and *V. sinensis*.

### 3.5 Differences of related biological processes between *R. sinicus* and *V. sinensis* revealed by DAMs

**FIGURE 6:**
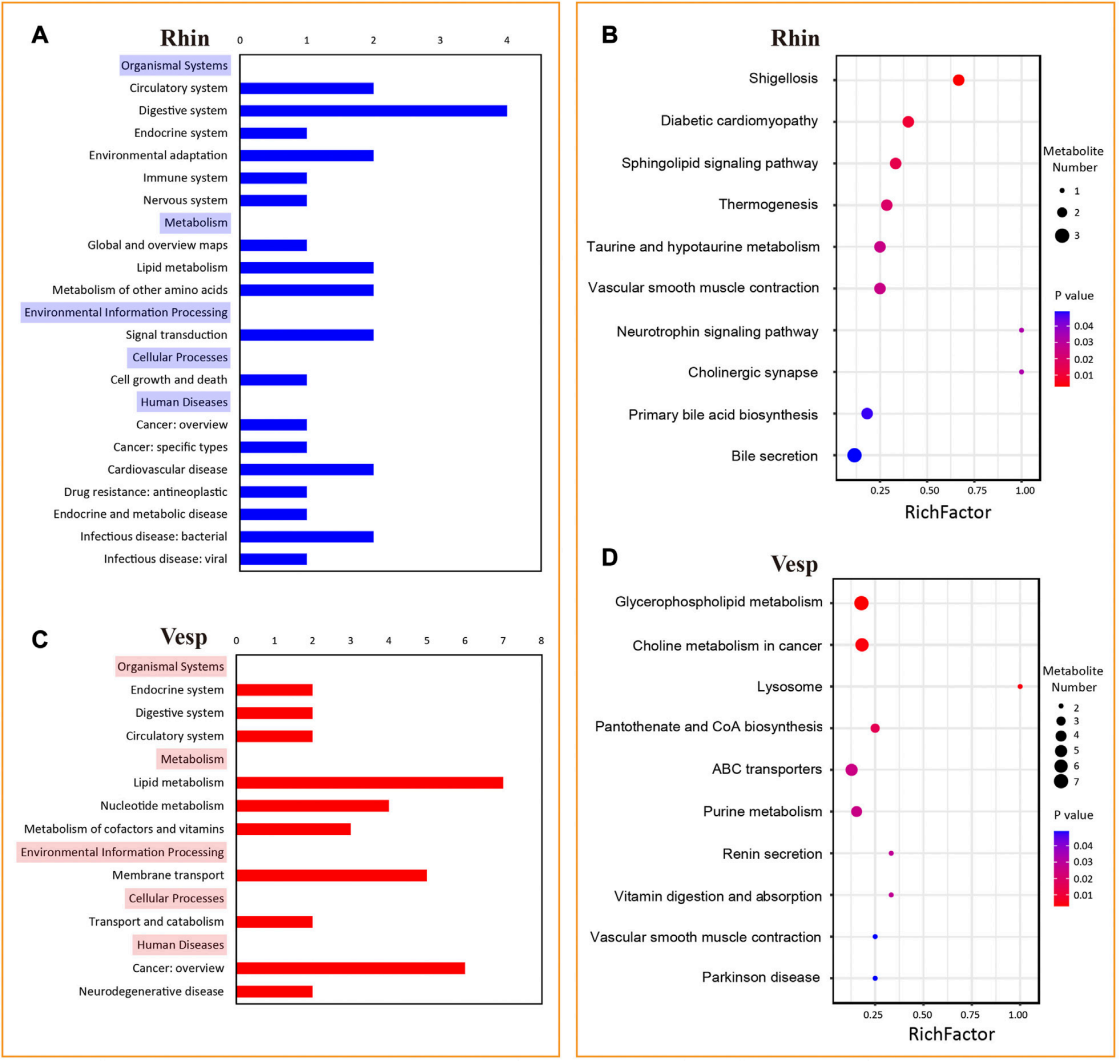
KEGG pathways significantly associated with 129 and 228 DAMs with significantly abundant levels in the cochleae of *R. sinicus* and *V. sinensis*, respectively. **(A)** and **(C)** KEGG A and B categories significantly associated with DAMs for *R. sinicus* and *V. sinensis*. **(B)** and **(D)** The top 10 KEGG pathways significantly associated with DAMs for *R. sinicus* and *V. sinensis*.

Numerous KEGG pathways were significantly associated with the DAMs from the *R. sinicus* versus *V. sinensis* comparison (Figure 6). In detail, 39 pathways were significantly associated with the DAMs predominantly accumulated in *R. sinicus*, while 10 pathways were for the DAMs predominantly accumulated in *V. sinensis*. According to the order of *p*-values, the top 10 pathways are represented in a bubble plot (Figures 6B, D), which indicates that different biological processes were activated in the cochleae of *R. sinicus* and *V. sinensis*. In particular, two nervous system-related pathways, Neurotrophin signaling pathway (ko04722) and Cholinergic synapse (ko04725), were significantly associated with the DAMs predominantly accumulated in *R. sinicus*, both of which are closely associated with the process of auditory perception. Several signal transduction-related pathways were also found to be important in the cochlea of *R. sinicus*, such as the MAPK signaling pathway (ko04010), ErbB signaling pathway (ko04012), Ras signaling pathway (ko04014), Rap1 signaling pathway (ko04015), and Calcium signaling pathway (ko04020).

**FIGURE 7:**
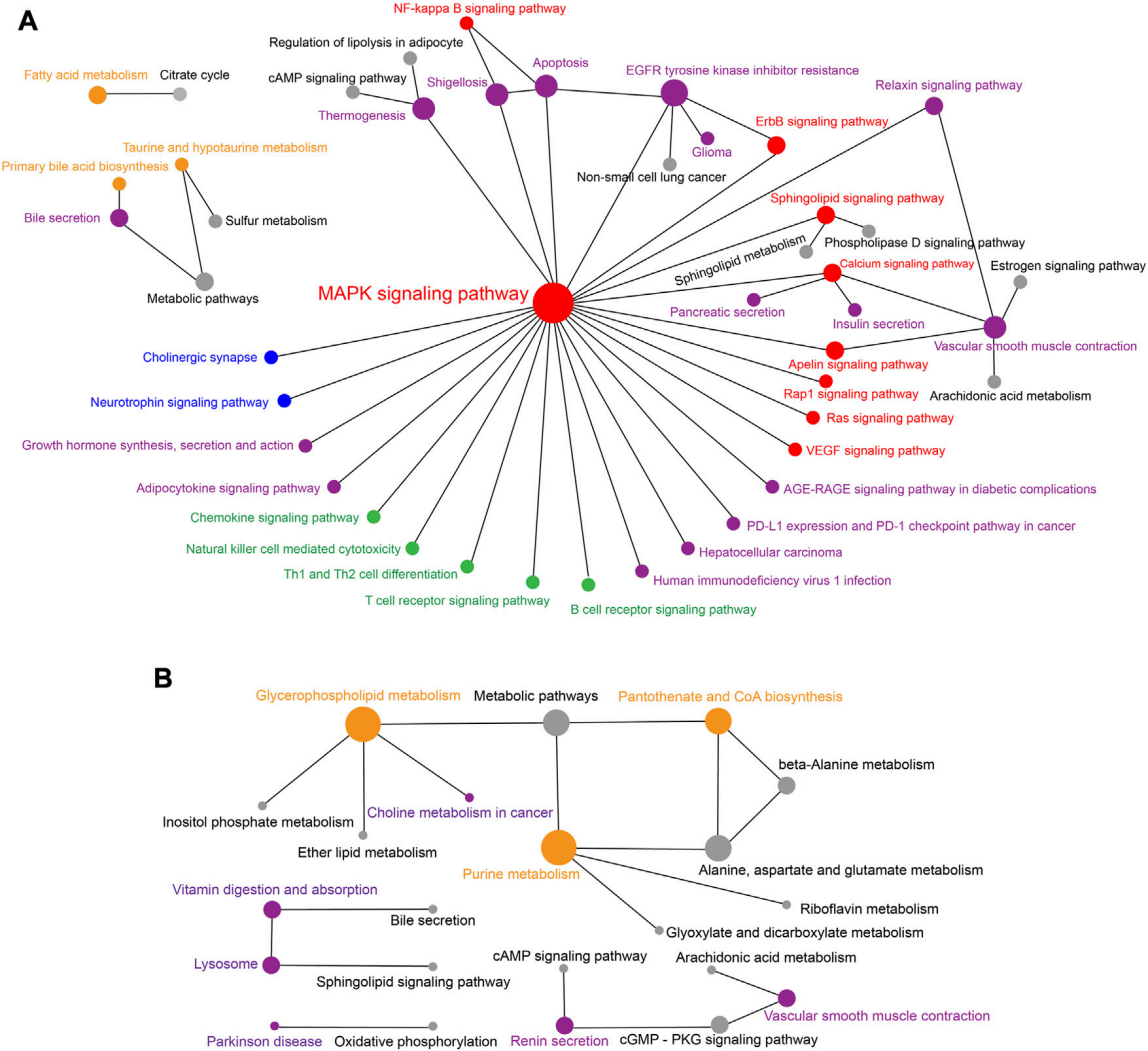
Network relationships of the KEGG pathways significantly associated with the significantly abundant metabolites in the cochlea of *R. sinicus* **(A)** and *V. sinensis* **(B)**, respectively. The size of the dot indicates the level of connectivity of a specific pathway interacting with others. Significantly enriched pathways are indicated by colored dots rather than gray ones. Dot and font colors are as follows: signal transduction-related pathways (red), metabolism-related pathways (orange), nervous system-related pathways (blue), immune system-related pathways (green), and others (purple).

Furthermore, network analysis for significantly enriched KEGG pathways was performed to obtain insights into the relationships of the metabolites differently accumulated in *R. sinicus* and *V. sinensis*. The network for *R. sinicus* as shown in Figure 7A indicated that the MAPK signaling pathway (ko04010) was the most important core pathway, which communicated with multiple other signaling pathways, such as the NF-kappa B signaling pathway (ko04064) and Calcium signaling pathway (ko04020). At the same time, it also connected with various biological systems, such as Nervous system, Immune system, and Endocrine system. Meanwhile, specific metabolism-related pathways were potentially more active in the cochlea of *V. sinensis*, as revealed by the network analysis of significantly enriched KEGG pathways (Figure 7B), including Glycerophospholipid metabolism (ko00564), Pantothenate and CoA biosynthesis (ko00770), and Purine metabolism (ko00230), which closely interacted with each other.

## 4 Discussion

Echolocating bats have attracted much attentions for their remarkable high-frequency hearing ability (Moss, 2018; Cao et al., 2022). However, there seems to be a lack of further understanding of the extensive metabolites in the key auditory organs. Among multiple omics approaches, metabolomics has gradually become the most direct and efficient method to explore complex biological traits (Fiehn, 2022). It is very necessary to identify the cochlear metabolites and to provide further scientific clues for understanding the molecular mechanisms of high-frequency hearing in echolocating bats. Therefore, untargeted metabolomics were performed here to obtain an insight into the various metabolites and biological processes in the cochleae of echolocating bats for the first time, and also to reveal the differences of metabolites and related physiological processes in the cochleae of the two bat species.

The cochleae of *R. sinicus* and *V. sinensis* were characterized by abundant metabolites that reflect specific biochemical pathways involved in the process of auditory perception. Metabolites classified as amino acids, peptides, and analogues were the most abundant in the cochleae of echolocating bats, while similar abundance levels were also detected in the inner ear fluid of guinea pig (Fujita et al., 2015; Pirttilä et al., 2019). Besides, amino acid metabolites were also detected in the inner ear of mice (Ji et al., 2019). In addition to amino acids, various metabolites, including hydroxy acids, carbohydrates, alcohols and polyols, homogeneous non-metal compounds, carboxylic acids, fatty acids, and purines and purine derivatives were detected in the inner ear fluid of guinea pig cochlea by gas chromatography–mass spectrometry (GC-MS) (Fujita et al., 2015), which were also detected in the cochleae of the two echolocating bats in this study by LC-MS. However, 4,717 annotated metabolites were detected in the cochlea of bats, which was a greater number than the 77 kinds of metabolites detected in the inner ear fluid of guinea pig cochlea (Fujita et al., 2015). This is reasonable given that the cochlea contains more microscopic structures and more abundant cells than the inner ear fluid of the cochlea, so the metabolites in the cochlea could be more abundant than those only detected in the inner ear fluid of the cochlea. Besides, various metabolites across the major metabolic pathways in central carbon metabolism, including amino acids, nucleotides, cytosine, L-methionine, L-arginine, glutamate, xanthurenic acid, aspartate, phenylalanine, tyrosine, aromatic amino acids, adenosine, oxidized glutathione, methionine, and tryptophan, were detected in the inner ear of mice, which may be involved in responses to noise trauma (Ji et al., 2019); those metabolites were also identified in the cochlea of our two echolocating bat species, which may also constitute the molecular basis to response to noise stimulation, for bats were more usually expose to high intensity noise environment.

In addition, more abundant metabolites were found in the cochlea of laryngeally echolocating bats, which are involved in various aspects of the physiological function of the cochlea, including metabolic processes of amino acids, carbohydrates, lipids, cofactors and vitamins, and nucleotides, as well as the immune system, nervous system, sensory system, membrane transport, and signal transduction. Previous studies demonstrated that hearing acquisition relies on the functional maturation and appropriate organization of the cochlea that couples the transfer of signaling, ions, and nutrients (Kelly and Chen, 2009; Ceriani and Mammano, 2012). Therefore, metabolites detected in the cochleae of bats that participated in the pathways related to various signal transductions, the nervous system, membrane transport, and transport and catabolism, among others, potentially provide comprehensive references for future studies on cochlear function.

Significant metabolic differences were detected between the cochleae of *R. sinicus* and *V. sinensis*. Compared with the levels in *V. sinensis*, 129 metabolites were identified to be significantly abundant in the cochlea of *R. sinicus*. Multiple pathways were shown to be influenced by these metabolites, including those involving signal transduction, the nervous system, endocrine system, lipid metabolism, and environmental adaptation. This indicates that these biological processes were different and specific in the cochlea of *R. sinicus*. Neurotrophin signaling pathway (ko04722) and Cholinergic synapse (ko04725) were the two most important pathways, which may play important roles in the auditory perception in *R. sinicus*. Neurotrophins have been identified as a key factor in the maintenance of spiral ganglion health, playing important roles in the normal function of cochleae (Shew et al., 2021). Previous studies identified several key features of cholinergic synapses in the cochleae of mammals, especially the efferent cholinergic synaptic transmission in the vestibular periphery (Poppi et al., 2020). Our findings suggest that the metabolites participating in the neurotrophin signaling pathway and cholinergic synapses are potentially crucial metabolic bases for the process of auditory perception in *R. sinicus*. As a representative species of CF bats, *R. sinicus* possessed unique and high-frequency CF component in their echolocation calls compared with *V. sinensis*, this regarded as one of the possible reasons for the more abundant neurotrophin signaling pathway and cholinergic synapses in the cochleae of *R. sinicus.* Besides, CF bat have developed special cochlear adaptations, such as auditory fovea and neurons with extraordinarily sharp frequency tuning, these may consist of other potential reasons for the differences between *R. sinicus* and *V. sinensis* revealed by DAMs and related pathways. Besides, it has been reported that SK2 calcium-activated potassium channel is required for cholinergic function in mouse cochlear hair cells (Kong et al., 2008; Jordan et al., 2013) and also for the long-term maintenance of efferent synapses on-to mammalian cochlear hair cells (Murthy et al., 2009). A previous study demonstrated that the *SK2* gene, as one of the most important hearing-related genes, has undergone more rapid evolution in echolocating mammals than in non-echolocating mammals and may be involved in the high-frequency hearing of echolocating mammals (Wang et al., 2021). Therefore, adaptive evolutionary changes detected in the cochlea of *R. sinicus* may be revealed by analyses of both metabolites and genes, and even complex networks of interactions between them, which have adapted in the process of auditory perception.

Notably, compared with the findings in FM bat, different nervous system activities were also demonstrated in the cochlea of *R. sinicus* (CF bat), as revealed by the gene expression data obtained using RNA-Seq (Wang et al., 2018). Genes overexpressed in *R. sinicus* (CF bat) compared with the levels in *Taphozous melanopogon* (FM bat) were shown to be significantly associated with various nervous system components, such as Cholinergic synapse (ko04725), Glutamatergic synapse (ko04724), and Dopaminergic synapse (ko04728). Similarly, at the metabolic level here, two nervous system-related pathways, Cholinergic synapse (ko04725) and Neurotrophin signaling pathway (ko04722) were significantly associated with the DAMs predominantly accumulated in *R. sinicus*. The Cholinergic synapse (ko04725) was the same pathway that significantly enriched by overexpressed genes and high-accumulated metabolites detected in the cochleae of *R. sinicus*. Taken together, both expressed genes and metabolites detected in the cochlea of *R. sinicus* indicated that Cholinergic synapse (ko04725) is an important physiological basis underlying the auditory function. However, more studies are needed to conduct to verify if these clues were suitable for other CF bats.

In addition, the activity of multiple signal transduction pathways was also detected in the cochlea of *R. sinicus* compared with the findings for *V. sinensis*, which suggested more active signal transduction in the former. Importantly, the MAPK signaling pathway (ko04010) was identified as the most central signal transduction pathway. Previously, metabolomic and bioinformatic analyses indicated that this is the major pathway in various types of hearing loss (Alagramam et al., 2014; Muurling and Stankovic, 2014; Liu et al., 2021). Numerous studies related to hearing damage have also shown that noise exposure immediately activates the cochlear MAPK signaling pathway, which plays important roles in maintaining normal physiological function of the cochlea (Maeda et al., 2013; Alagramam et al., 2014; Kurabi et al., 2017). For nocturnal echolocating bats, which are highly dependent on acoustic signals and are continually exposed to ultrasonic signals in daily life, the MAPK signaling pathway could be particularly important in regulating the development and survival of auditory hair cells. Compared with *V. sinensis* (around 24.2 kHz), *R. sinicus* (around 83.15 kHz) has developed a much higher- and constant-frequency component in its acoustic signals (Fukui et al., 2004; Xie et al., 2017; Wang et al., 2018), so the metabolites significantly accumulated in the MAPK signaling pathway may constitute an important auditory basis in the cochlea of *R. sinicus*. The molecular interactions of the MAPK signaling pathway and other signaling pathways play an important role in the survival of hair cells and the normal function of cochlea. Pathways such as the calcium signaling pathway play numerous fundamental roles in the inner ear (including in neurotransmitter release and synaptic transmission) (Ceriani and Mammano, 2012; Liu et al., 2021), along with multiple signaling pathways that interact with the MAPK signaling pathway in the cochlea of *R. sinicus*. Similarly, among the pathways significantly associated with the overexpressed genes detected in the cochlea of *R. sinicus* rather than in *T. melanopogon*, signal transduction pathways were almost the most represented, second only to nervous system pathways (Wang et al., 2018). Taken together, evidence from comparative cochlear transcriptomic and metabolomic analyses consistently indicated that signal transduction might be more active in the cochlea *R. sinicus* compared with FM bats.

More abundant metabolites in the cochlea of *V. sinensis* when compared with the levels in *R. sinicus* were significantly associated with metabolic pathways including Glycerophospholipid metabolism (ko00564), Pantothenate and CoA biosynthesis (ko00770), and Purine metabolism (ko00230), which have generally been demonstrated to be involved with exposure to noise. A recent animal experimental study on the brain of rats found that purine metabolism was markedly altered by acoustic trauma (He et al., 2017). Furthermore, in mice, purinergic signaling has been suggested to protect against noise trauma and contribute to cochlear adaptation to elevated sound levels (Muñoz et al., 1999; Housley et al., 2013). It was also identified that metabolites that showed a significant difference in accumulation between microtia and healthy ear cartilage were associated with Pantothenate and CoA biosynthesis, so these may have an association with the development of microtia ear cartilage (Yang and He, 2020). Finally, with regard to Glycerophospholipid metabolism, this has potential protective roles in the cochlea, which was reported to be significantly related to noise-induced hearing loss (Xie et al., 2021). Taken together, our results indicated that different metabolic processes presenting in the cochleae of *R. sinicus* and *V. sinensis*. The specific metabolic pathways identified in *V. sinensis* could be related to cochlear development and protective mechanisms in response to echolocation calls and echoes. Moreover, in this study, it should be noted that the *V. sinensis* individuals were collected from a large colony roosting under a bridge, containing about 10,000 individuals living together from early June to late September each year. These bats are thus exposed to high-intensity noise from their peers at night and from traffic in the day (Song et al., 2020). The specific metabolic pathways detected in the cochlea of *V. sinensis* could also be related to cochlear development and protective mechanisms in response to inhabiting a noisy environment. However, more evidences are needed in future to uncover the functions of these metabolic pathways in the cochlea of *V. sinensis*.

To better understand the molecular bases underlying high-frequency hearing of bats, our study provided an overview of the cochlear metabolites and associated biological processes of two typical laryngeally echolocating bat species. Our study takes new insights into the molecular mechanisms of auditory perception of echolocating bats. In consideration of the limitations of single omics, future work should focus on a comprehensively study to fully uncover the molecular mechanisms and key network relationships combing the transcriptome, proteome, and metabolome in echolocating bats. Unfortunately, no sufficient bat cochlear samples were left here for the experimental validation which needs to be further studied. Therefore, comparative cochlear metabolic and transcriptomic sequencing of more representative bat species from CF, FM and even non-echolocating bat groups will be further performed in future along with the experimental validations to reveal the genetic bases of high-frequency hearing and also the different molecular bases and biological mechanisms underlying the auditory processes of bats with distinct hearing traits.

## Data Availability

The datasets presented in this study can be found in the article/Supplementary Material.
