# Supplementary material for: Untargeted metabolomics of the cochleae from two laryngeally echolocating bats
Source: Front Mol Biosci. 2023 Apr 19;10:1171366. doi: 10.3389/fmolb.2023.1171366 (PMC10154556; doi:10.3389/fmolb.2023.1171366)
Supplement: Supplementary file 1 [file DataSheet1.ZIP › Supplementary Material/Supplementary Material.pdf]

Supplementary Material

Untargeted metabolomics of the cochleae from two laryngeally echolocating bats

Hui Wang*, Ruyi Sun, Ningning Xu, Xue Wang, Mingyue Bao, Xin Li, Jiqian Li, Aiqing Lin and Jiang Feng*

*** Correspondence:** Corresponding Authors: wangh681@nenu.edu.cn, fengj@nenu.edu.cn.

1 Supplementary Figures and Tables

For more information on Supplementary Material and for details on the different file types accepted, please see here.

1.1 Supplementary Figures

Figure S1. Total ion chromatograms (TIC) of all samples. (**A**) indicates positive ion chromato-grams and (**B**) indicates negative ion chromatograms. In the legend, QC, Rhin, and Vesp stand for quality control, *R. sinicus*, and *V. sinensis* samples, respectively. These abbreviations are also used elsewhere in this paper.

Figure S2. Super-classification of DAMs detected in the cochleae of *R. sinicus* and *V. sinensis*.

Figure S3. Classification of DAMs detected in the cochleae of *R. sinicus* and *V. sinensis*. (**A**) Venn diagram of the Classifications and DAMs. The numbers in parentheses represent the number of DAMs. (**B**) The common classifications of DAMs. (**C**) Unique classifications of DAMs detected in *R. sinicus* and *V. sinensis*.

Figure S4. Sub-classification of DAMs detected in the cochleae of *R. sinicus* and *V. sinensis*. (**A**) Venn diagram of the Sub-classifications and DAMs. The numbers in parentheses represent the numbers of DAMs. (**B**) The common sub-classifications of DAMs. (**C**) Unique sub-classifications of DAMs detected in *R. sinicus* and *V. sinensis*.

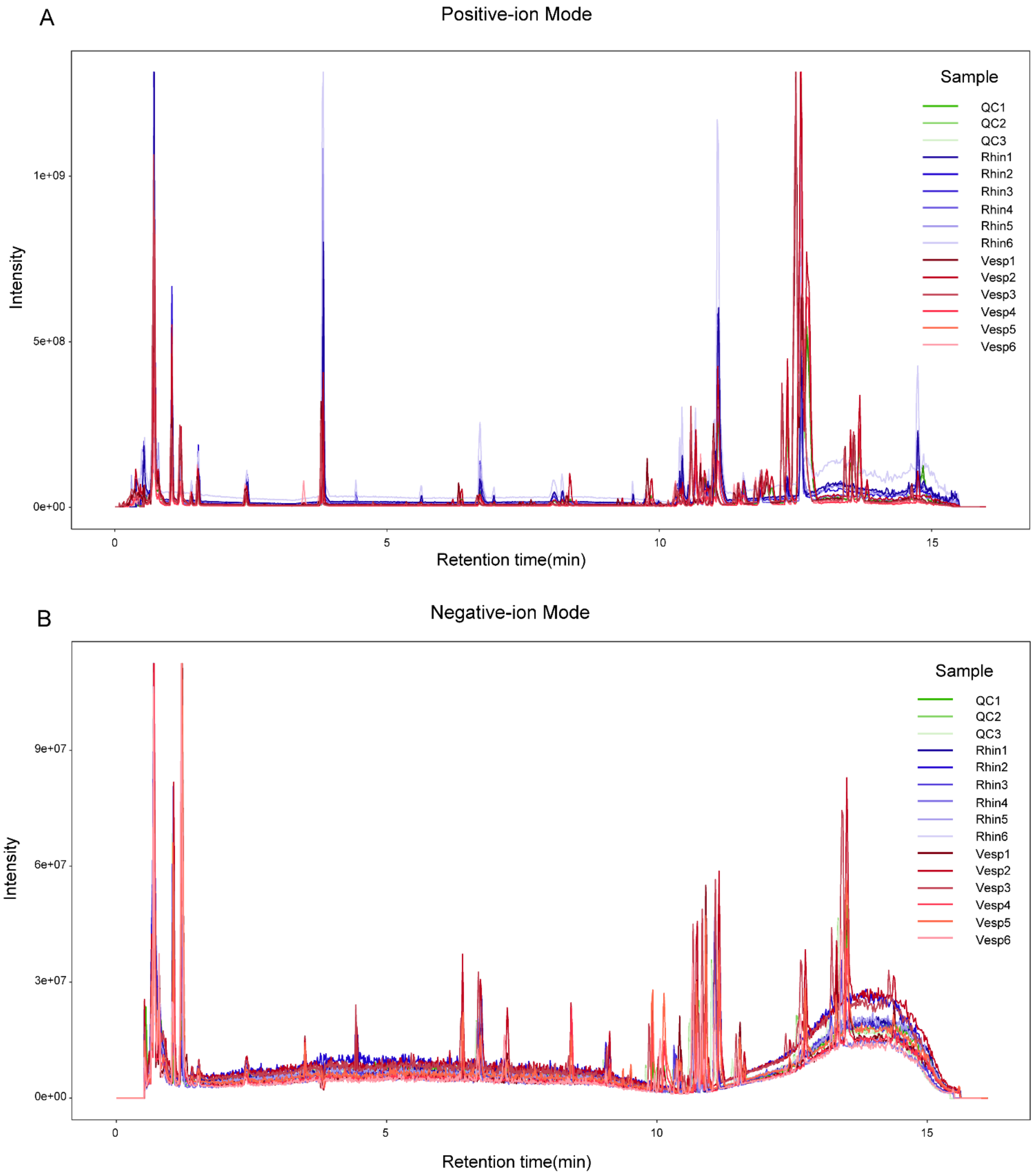

Figure S1. Total ion chromatograms (TIC) of all samples. (**A**) indicates positive ion chromato-grams and (**B**) indicates negative ion chromatograms. In the legend, QC, Rhin, and Vesp stand for quality control, *R. sinicus*, and *V. sinensis* samples, respectively. These abbreviations are also used elsewhere in this paper.

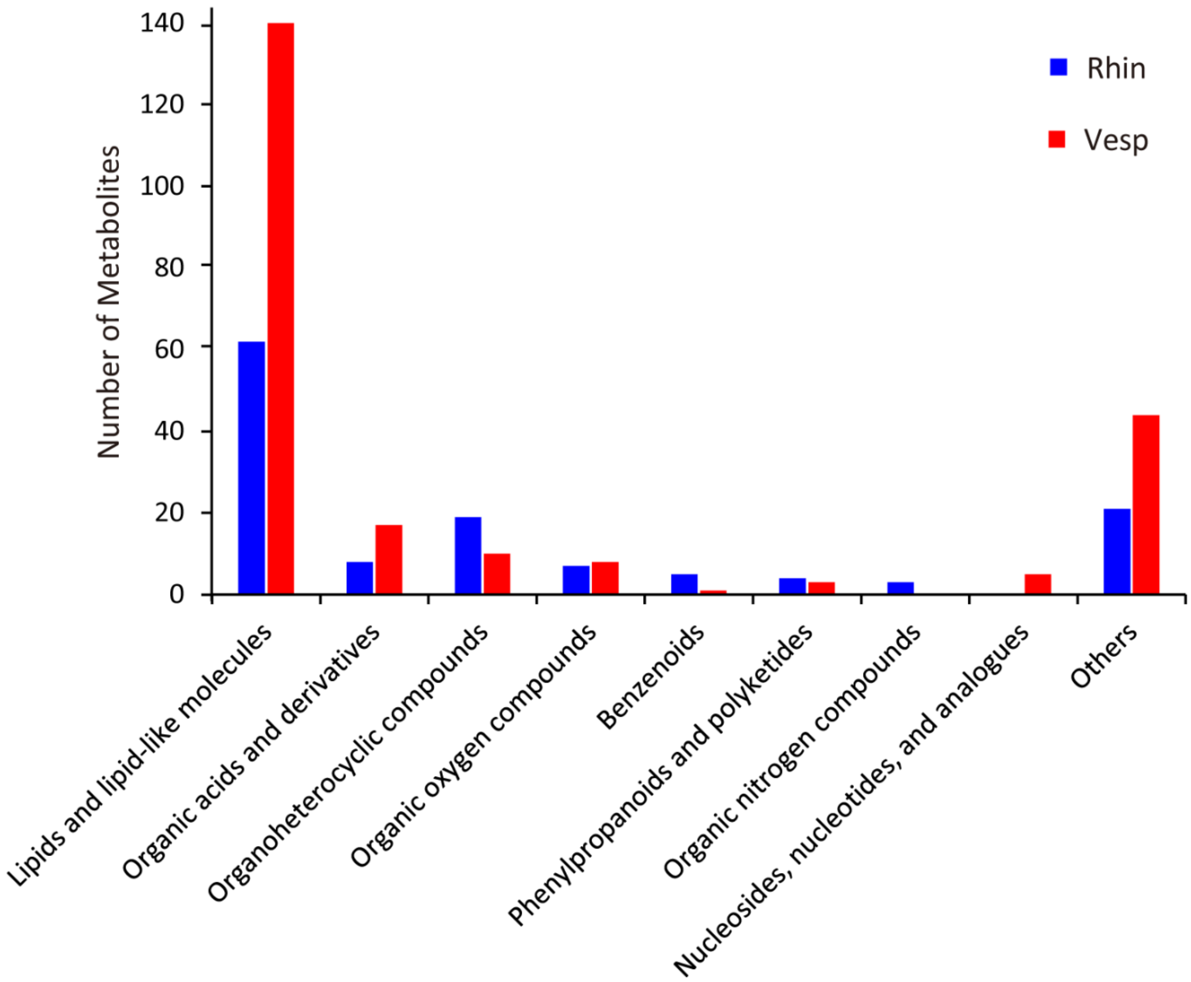

Figure S2. Super-classification of DAMs detected in the cochleae of *R. sinicus* and *V. sinensis*.

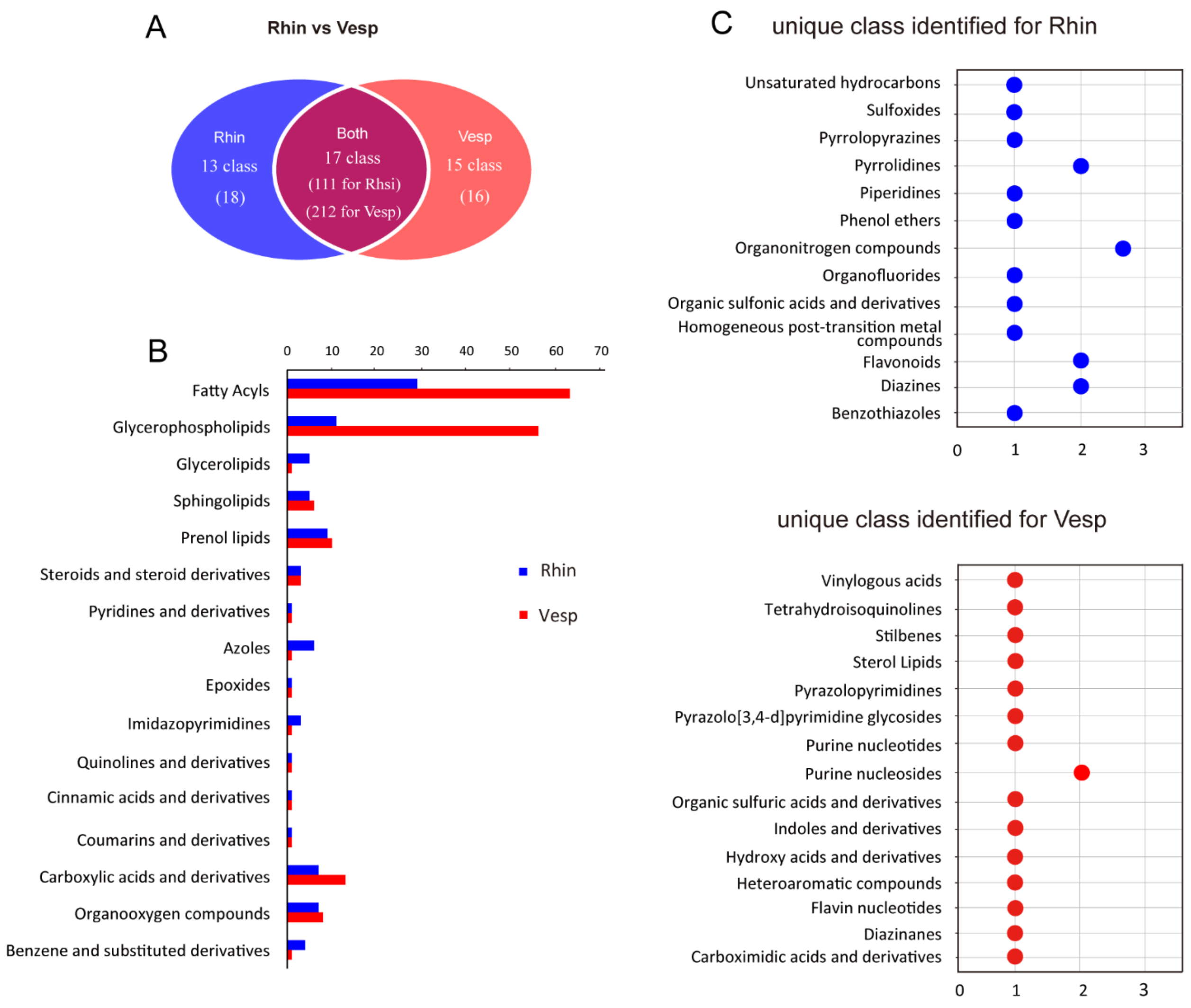

Figure S3. Classification of DAMs detected in the cochleae of *R. sinicus* and *V. sinensis*. (**A**) Venn diagram of the Classifications and DAMs. The numbers in parentheses represent the number of DAMs. (**B**) The common classifications of DAMs. (**C**) Unique classifications of DAMs detected in *R. sinicus* and *V. sinensis*.

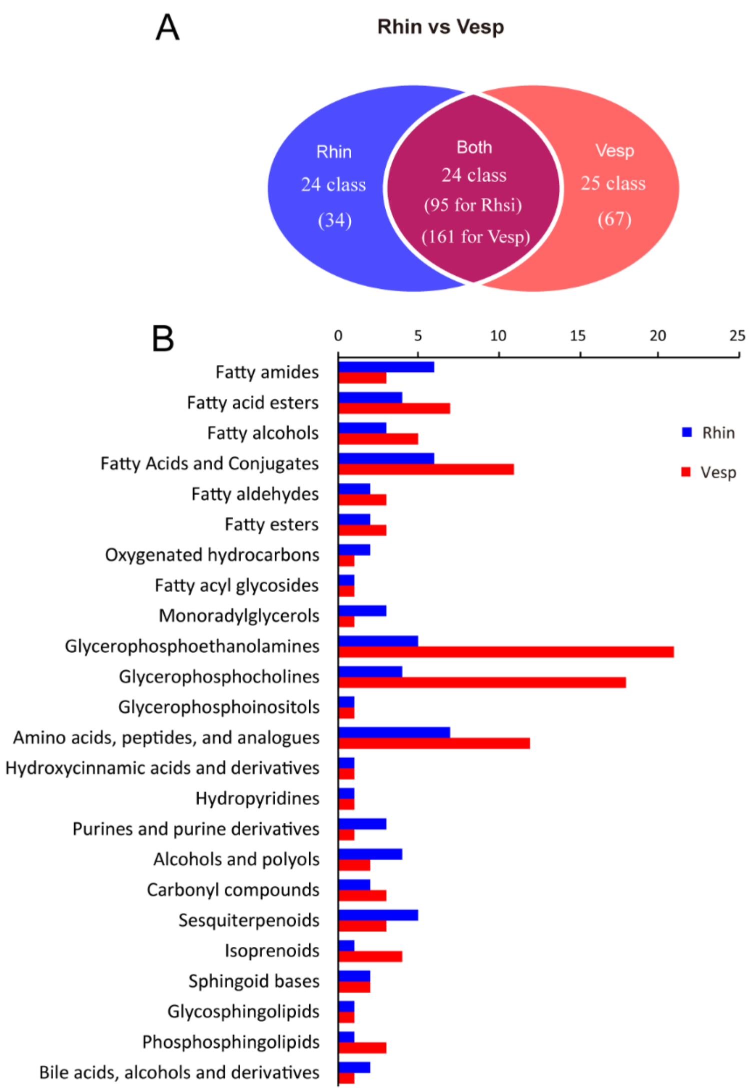

C

Unique sub-class identified for Rhin	Number
Oxazoles	4
Hydrocarbons	3
Benzoic acids and derivatives	2
Diradylglycerols	2
Flavonoid glycosides	2
Monoterpenoids	2
N-acylpyrrolidines	2
Amines	1
Cyclopyrrolones	1
Ethers	1
Glycerophosphoglycerols	1
Imidazoles	1
N-acylpiperidines	1
Organosulfonic acids and derivatives	1
Other Sphingolipids	1
Oximes	1
Phenylacetamides	1
Pyrazines	1
Pyrimidines and pyrimidine derivatives	1
Quaternary ammonium salts	1
Quinone and hydroquinone lipids	1
Steroid lactones	1
Thiazoles	1
Unsaturated aliphatic hydrocarbons	1

Unique sub-class identified for Vesp	Number
Eicosanoids	16
Octadecanoids	10
Oxidized glycerophospholipids	10
Glycerophosphoserines	4
Carbohydrates and carbohydrate conjugates	3
Lineolic acids and derivatives	3
Diterpenoids	2
Glycerophosphates	2
Androstane steroids	1
Benzenesulfonamides	1
Beta hydroxy acids and derivatives	1
Bile acids and derivatives	1
Carboximidic acids	1
Cholestane steroids	1
Indolyl carboxylic acids and derivatives	1
Oxirane carboxylic acids and derivatives	1
Piperazines	1
Purine ribonucleotides	1
Pyranocoumarins	1
Pyrazoles	1
Pyrazolo[3,4-d]pyrimidines	1
Quinoline carboxylic acids	1
Sulfuric acid esters	1
Tricarboxylic acids and derivatives	1
Triterpenoids	1

Figure S4. Sub-classification of DAMs detected in the cochleae of *R. sinicus* and *V. sinensis*. (**A**) Venn diagram of the Sub-classifications and DAMs. The numbers in parentheses represent the numbers of DAMs. (**B**) The common sub-classifications of DAMs. (**C**) Unique sub-classifications of DAMs detected in *R. sinicus* and *V. sinensis*.

1.2 Supplementary Tables

Table S1. Detailed information of all identified cochlear metabolites of the two laryngeally echolocating bats.

Table S2. Detailed information of cochlear metabolites identified at Super-class, Class, and Sub-class levels (Total, pos-ion and neg-ion).

Table S3. KEGG pathways identified for all cochlear metabolites.
