# Supplementary material for: Untargeted metabolomics of the cochleae from two laryngeally echolocating bats
Source: Front Mol Biosci. 2023 Apr 19;10:1171366. doi: 10.3389/fmolb.2023.1171366 (PMC10154556; doi:10.3389/fmolb.2023.1171366)
Supplement: Supplementary file 1 [file DataSheet1.ZIP › Supplementary Material/Supplementary_Material.docx]

Supplementary Material

Untargeted metabolomics of the cochleae from two laryngeally echolocating bats

Hui Wang*, Ruyi Sun, Ningning Xu, Xue Wang, Mingyue Bao, Xin Li, Jiqian Li, Aiqing Lin and Jiang Feng*

*** Correspondence:** Corresponding Authors: [wangh681@nenu.edu.cn](mailto:wangh681@nenu.edu.cn), [fengj@nenu.edu.cn](mailto:fengj@nenu.edu.cn).

# Supplementary Figures and Tables

For more information on Supplementary Material and for details on the different file types accepted, please see [here](https://www.frontiersin.org/guidelines/author-guidelines#supplementary-material).

## Supplementary Figures

**Figure S1.** Total ion chromatograms (TIC) of all samples. (**A**) indicates positive ion chromato-grams and (**B**) indicates negative ion chromatograms. In the legend, QC, Rhin, and Vesp stand for quality control, *R. sinicus*, and *V. sinensis* samples, respectively. These abbreviations are also used elsewhere in this paper.

**Figure S2**. Super-classification of DAMs detected in the cochleae of *R. sinicus* and *V. sinensis*.

**Figure S3**. Classification of DAMs detected in the cochleae of *R. sinicus* and *V. sinensis*. (**A**) Venn diagram of the Classifications and DAMs. The numbers in parentheses represent the number of DAMs. (**B**) The common classifications of DAMs. (**C**) Unique classifications of DAMs detected in *R. sinicus* and *V. sinensis*.

**Figure S4**. Sub-classification of DAMs detected in the cochleae of *R. sinicus* and *V. sinensis*. (**A**) Venn diagram of the Sub-classifications and DAMs. The numbers in parentheses represent the numbers of DAMs. (**B**) The common sub-classifications of DAMs. (**C**) Unique sub-classifications of DAMs detected in *R. sinicus* and *V. sinensis*.


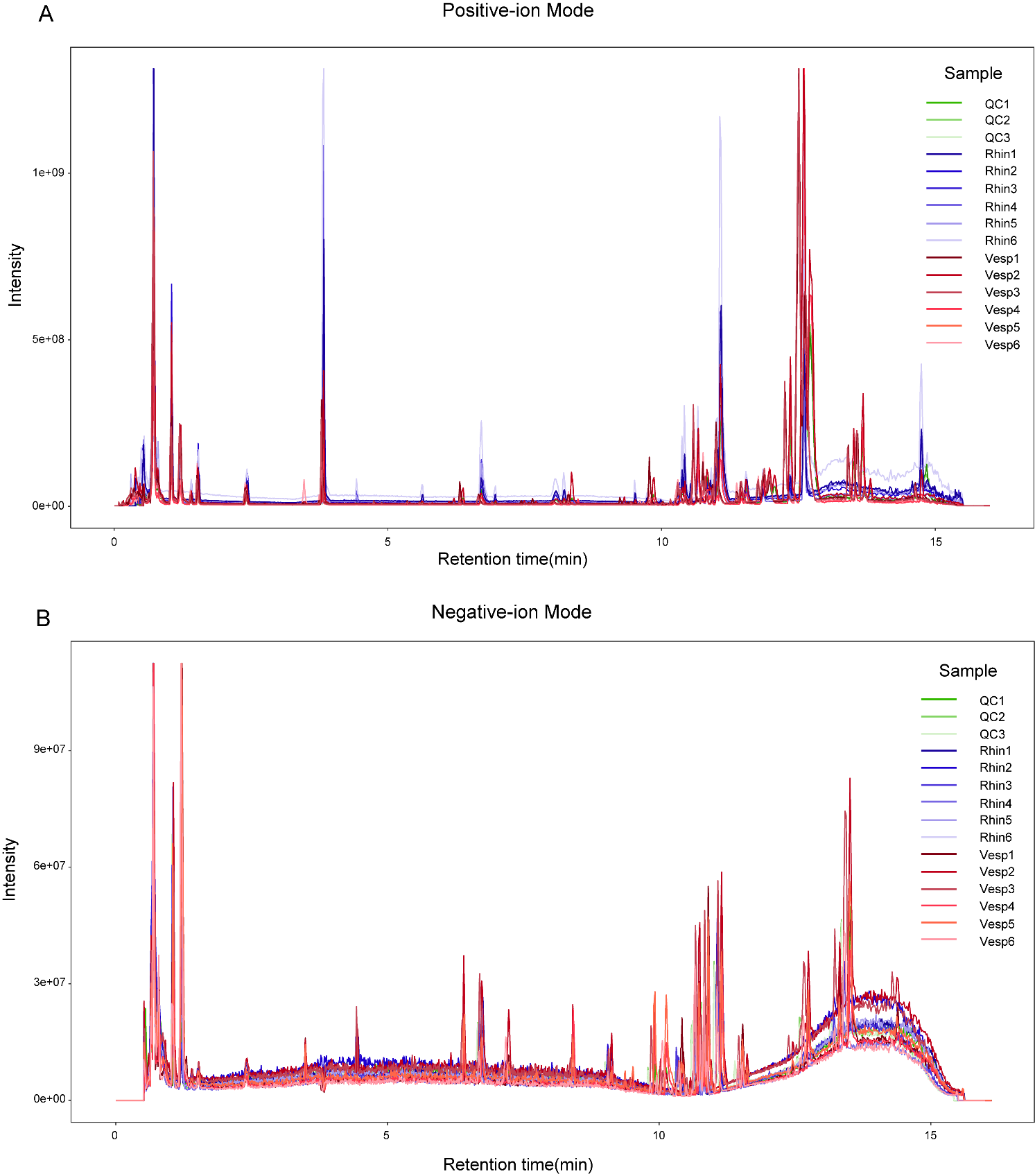


**Figure S1.** Total ion chromatograms (TIC) of all samples. (**A**) indicates positive ion chromato-grams and (**B**) indicates negative ion chromatograms. In the legend, QC, Rhin, and Vesp stand for quality control, *R. sinicus*, and *V. sinensis* samples, respectively. These abbreviations are also used elsewhere in this paper.


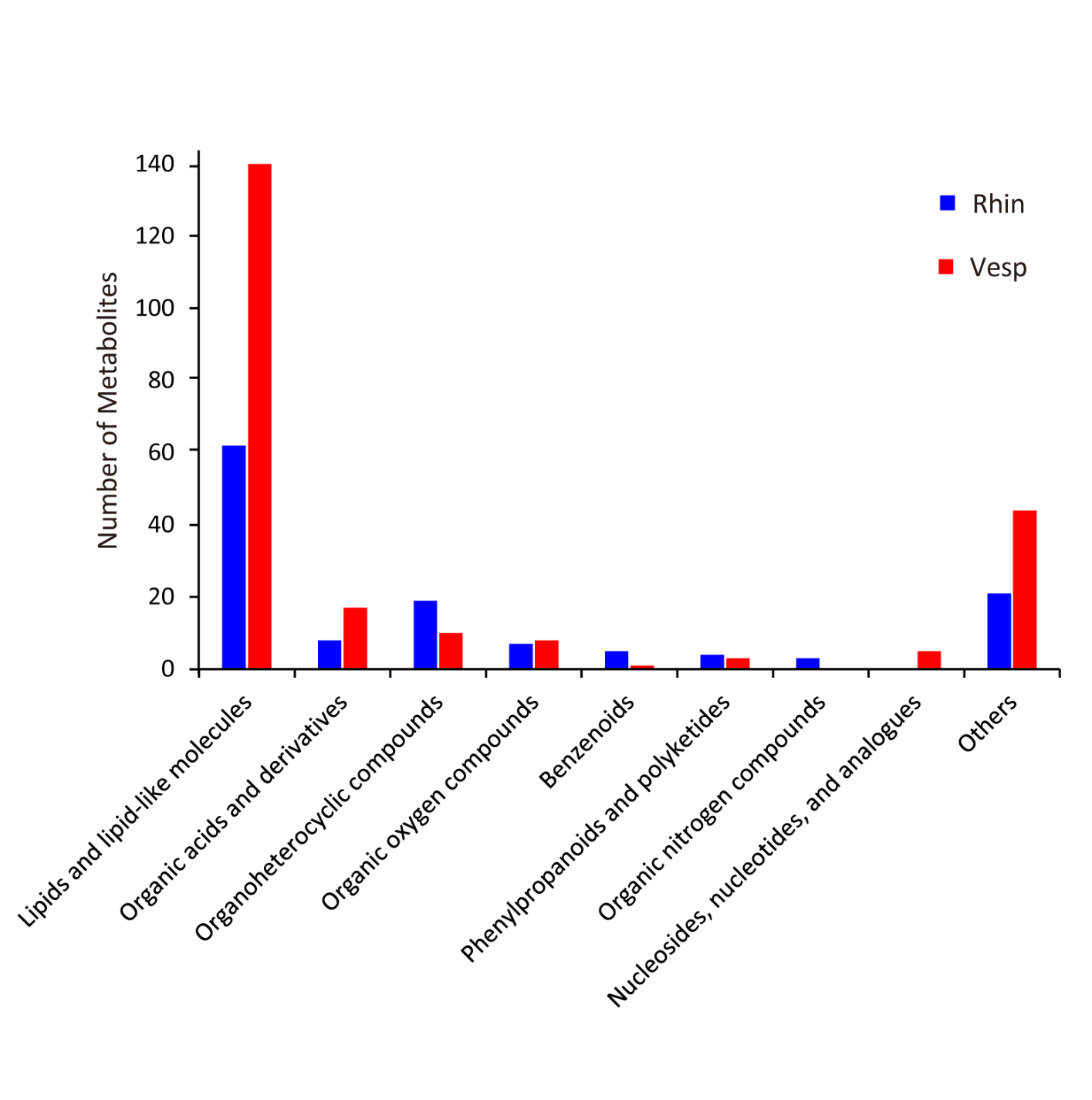


**Figure S2**. Super-classification of DAMs detected in the cochleae of *R. sinicus* and *V. sinensis*.


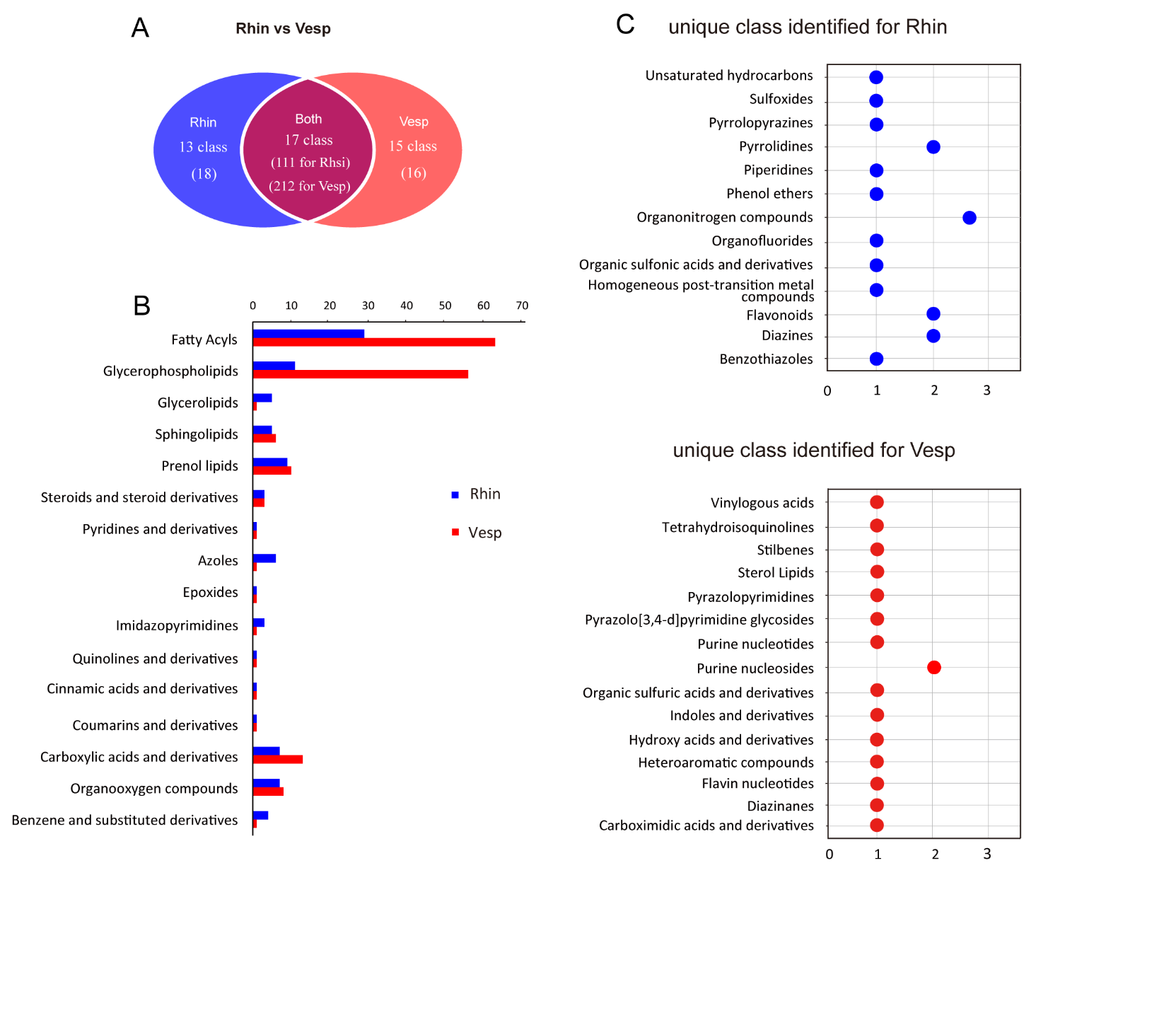


**Figure S3**. Classification of DAMs detected in the cochleae of *R. sinicus* and *V. sinensis*. (**A**) Venn diagram of the Classifications and DAMs. The numbers in parentheses represent the number of DAMs. (**B**) The common classifications of DAMs. (**C**) Unique classifications of DAMs detected in *R. sinicus* and *V. sinensis*.


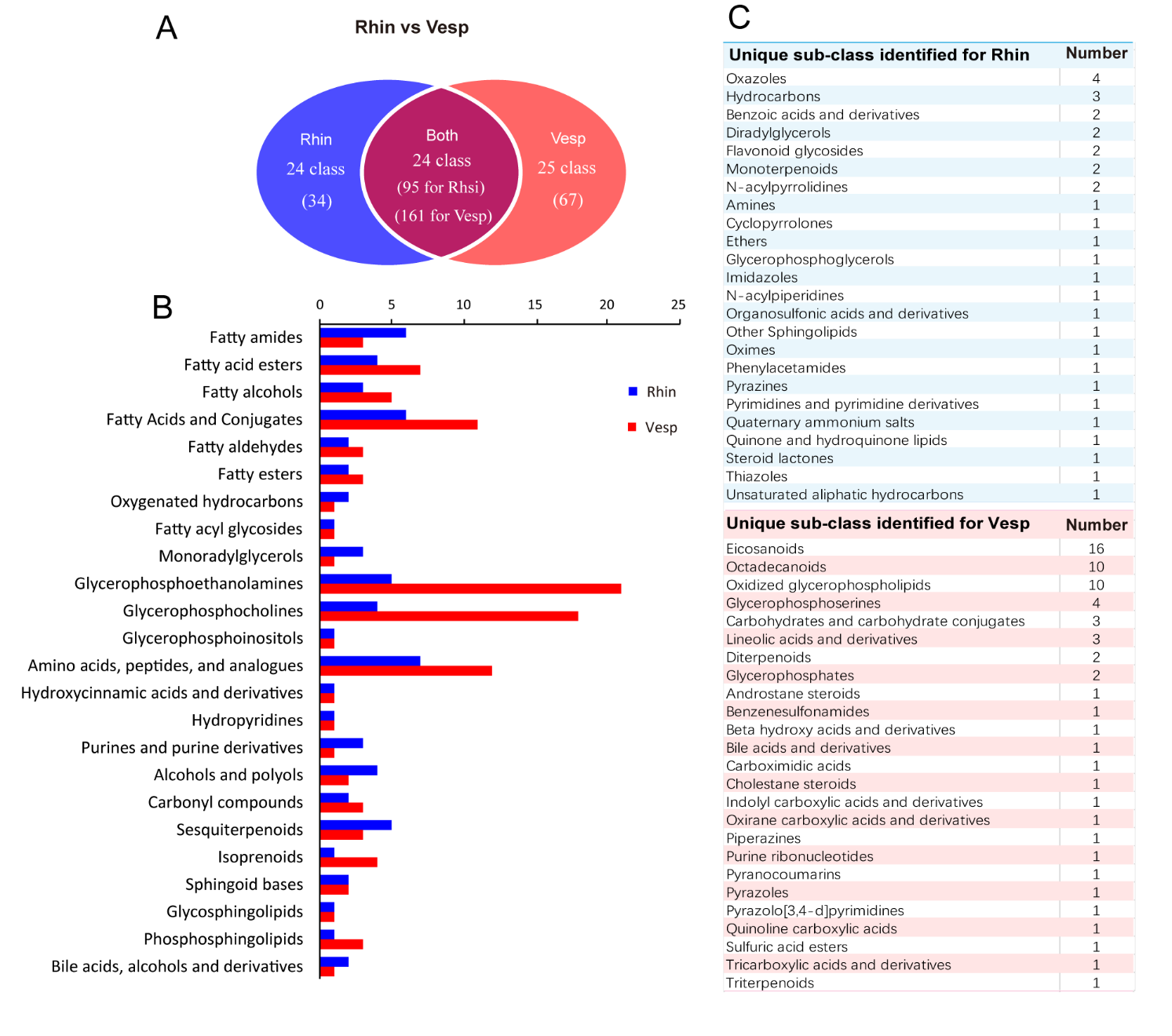


**Figure S4**. Sub-classification of DAMs detected in the cochleae of *R. sinicus* and *V. sinensis*. (**A**) Venn diagram of the Sub-classifications and DAMs. The numbers in parentheses represent the numbers of DAMs. (**B**) The common sub-classifications of DAMs. (**C**) Unique sub-classifications of DAMs detected in *R. sinicus* and *V. sinensis*.

## Supplementary Tables

**Table S1**. Detailed information of all identified cochlear metabolites of the two laryngeally echolocating bats.

**Table S2**. Detailed information of cochlear metabolites identified at Super-class, Class, and Sub-class levels (Total, pos-ion and neg-ion).

**Table S3**. KEGG pathways identified for all cochlear metabolites.
